# Fermented Foods: New Concepts and Technologies for the Development of New Products, Quality Control

**DOI:** 10.3390/foods11030441

**Published:** 2022-02-02

**Authors:** Marios Mataragas, Loulouda Bosnea

**Affiliations:** Dairy Research Department, Institute of Technology of Agricultural Products, Hellenic Agricultural Organization-DIMITRA, 3rd Ethnikis Antistaseos Str., 45221 Ioannina, Greece; louloudabosnea@gmail.com

Fermentation has been of great interest for humans since antiquity and has been extensively used in all cultures worldwide. Well known fermentation products include beer, wine, bread, fermented vegetables, and cheeses. Fermentation is a dynamic process which is based on the microbial activity of lactic acid bacteria, yeasts, and/or filamentous fungi which significantly contribute to food preservation and the organoleptic characteristics of the products. Over the years, several methods have been developed and used in order to comprehend and improve fermentation processes. Lately, new approaches in food biotechnology along with approaches based on molecular microbiology and bioinformatics have had a major impact on both the study of fermented foods’ ecology and the development of healthier, safe, and high-quality food products. Genomics and other omics technologies in the next years will be irreplaceable tools for the assessment and monitoring of microbiological and physicochemical changes occurring in fermented foods. This Special Issue contains six research papers on several traditional fermentation products such as bread and sourdough, table olives, cheeses, beer, and fermented fish sauce and a review article on the effect of LAB bacteria in food safety and spoilage prevention [1,2,3,4,5,6,7]. In the respective research papers, the authors have used classic and advanced analytical methods as well as molecular techniques to evaluate the traditional and novel fermentation products or processes in an attempt to provide insight of fermentation and/or improve traditional fermented products.

More specifically, Ibrahim et al. reviewed the potential of lactic acid bacteria (LAB) and their metabolites as natural antimicrobial alternatives for the improvement of food safety and spoilage prevention. The review summarizes the mechanisms of action and applications of LAB for food spoilage prevention and foodborne disease control [1].

Since ancient times, bread has represented one of the pillars of cultural heritage and the major source of dietary carbohydrates. In modern bread making, baker’s yeast is the most used starter, but the use of sourdough goes hand in hand with modern consumers’ demand for tasteful leavened baked goods with extended shelf-life and improved nutritional and functional properties. In this frame, Molfeta et al. [2] aimed to assess the influence of a mixture of flours (wheat, barley, oat, rye, buckwheat) traditionally used in Europe for bread-making, as well as the influence of sourdough as baking improver on chemical and sensory features and the in vitro glycemic index and antioxidant activity of bread. In addition, Syrokou et al. [3] studied the microecosystem of 13 homemade spontaneously fermented wheat sourdoughs from different regions of Greece, 12 of which originate from regions not previously assessed through the combined use of molecular techniques such as culture-dependent (classical approach; clustering by Random Amplified Polymorphic DNA-Polymerase Chain Reaction (RAPD-PCR), identification by PCR species-specific for Lactiplantibacillus plantarum, and sequencing of the 16S-rRNA and 26S-rRNA gene for Lactic Acid Bacteria (LAB) and yeasts, respectively) and independent approaches (DNA- and RNA-based PCR-Denaturing Gradient Gel Electrophoresis (DGGE)). The results of the study improved our knowledge on the respective micro-communities of Greek sourdoughs significantly.

Pappa et al. [4] successfully produced two traditional cheeses from the area of Epirus, commonly known as Kashkaval of Pindos, a semihard ewe milk cheese, and Urda, a whey cheese that is produced from the derived whey from kashkaval production. The main objective was the standardization of the production method of those two cheeses by modernizing the traditional cheese making methods. Both cheeses were successfully produced, and the obtained data may lead to the industrialized production of both cheeses with standard characteristics according to the traditional recipes and improve their recognition beyond the region.

Greiner et al. [5] focused on creating a fermented condiment from *Carcinus maenas* to control the population of the invasive species and establish an economically feasible use of the crabs. Even though further optimization of the product and process is essential, the experimental crab sauce product was comparable in many respects to currently available commercial fish sauce products.

The work of Martín-Vertedor and collaborators [6] focused on another traditional fermentation product, edible olives. Their study reported that controlling the temperature of the brine improves the fermentation process and enhances some of the parameters related to table olive quality such as the final phenolic fingerprint of table olives of the Manzanilla de Sevilla and Manzanilla Cacereña varieties. In addition, they reported that Sevillian-style production processes can be applied to both Manzanilla de Sevilla and Manzanilla Cacereña green table olive varieties with successful results. Heat treatment combined with temperature monitoring during the fermentation of table olives constitutes an approach to enhance the quality of Spanish-style table olives and shorten the fermentation time with adverse temperature conditions.

Finally, beer is a fermentation product that is generally considered safe with regard to microbiological risk; however, it is susceptible to the production of biogenic amines that constitute a potential risk for human health. Nalazek-Rudnicka et al. [7] developed an analytical method based on derivatization with tosyl chloride and high-performance liquid chromatography-tandem mass spectrometry (HPLC-MS/MS) that was used to determine 17 BAs in samples of commercially available beers and to monitor the changes in concentration of several BAs throughout the fermentation process. The results of the study indicate that monitoring the total content of BAs is required due to the potential risk to human health.

Overall, the Special Issue presents articles that provide an extra insight on several traditional products and report on how modern methods and techniques can become our new tools for further comprehending such a dynamic process as fermentation.
